# Biodegradation of Tetralin: Genomics, Gene Function and Regulation

**DOI:** 10.3390/genes10050339

**Published:** 2019-05-06

**Authors:** Belén Floriano, Eduardo Santero, Francisca Reyes-Ramírez

**Affiliations:** 1 Universidad Pablo de Olavide, Departamento de Biología Molecular e Ingeniería Bioquímica, 41013 Seville, Spain; bflopar@upo.es; 2 Centro Andaluz de Biología del Desarrollo, CSIC, Universidad Pablo de Olavide, Junta de Andalucía, Departamento de Biología Molecular e Ingeniería Bioquímica, 41013 Seville, Spain; esansan@upo.es

**Keywords:** tetralin, *Sphingopyxis granuli* strain TFA, *Rhodococcus* sp. strain TFB, redox proteins, carbon catabolite repression

## Abstract

Tetralin (1,2,3,4-tetrahydonaphthalene) is a recalcitrant compound that consists of an aromatic and an alicyclic ring. It is found in crude oils, produced industrially from naphthalene or anthracene, and widely used as an organic solvent. Its toxicity is due to the alteration of biological membranes by its hydrophobic character and to the formation of toxic hydroperoxides. Two unrelated bacteria, *Sphingopyxis granuli* strain TFA and *Rhodococcus* sp. strain TFB were isolated from the same niche as able to grow on tetralin as the sole source of carbon and energy. In this review, we provide an overview of current knowledge on tetralin catabolism at biochemical, genetic and regulatory levels in both strains. Although they share the same biodegradation strategy and enzymatic activities, no evidences of horizontal gene transfer between both bacteria have been found. Moreover, the regulatory elements that control the expression of the gene clusters are completely different in each strain. A special consideration is given to the complex regulation discovered in TFA since three regulatory systems, one of them involving an unprecedented communication between the catabolic pathway and the regulatory elements, act together at transcriptional and posttranscriptional levels to optimize tetralin biodegradation gene expression to the environmental conditions.

## 1. Introduction

The ability of microorganisms to degrade a variety of environmental pollutants and to use them as growth substrates is a powerful tool to clean up hazardous contaminants that are causing irreversible damage to the biosphere. Many hydrocarbons such as most alkanes can be efficiently degraded by several microorganisms [1] and many aromatic compounds can serve as growth substrates for a large variety of bacteria [2]. On the other hand, cycloalkanes seem to be more persistent to microbial attack [3]. Tetralin (1,2,3,4-tetrahydronaphthalene) is a bicyclic molecule composed of an aromatic and an alicyclic ring. It is naturally found in different crude oil reservoirs and also produced for industrial purposes from naphthalene by catalytic hydrogenation or from anthracene by cracking. It is widely used as a degreasing agent and solvent for fats, resins and waxes, as a substitute for turpentine in paints, lacquers, and shoe polishes, and in the petrochemical industry in connection with coal liquefaction [3]. Like other cyclic hydrocarbons, tetralin is toxic due to its lipophilic character, which facilitates its accumulation within the biological membranes causing changes in their structure and function, and to the formation of hydroperoxides inside the cells [4]. Its hydrophobic character, which reduces tetralin bioavailability, along with the fact that concentrations greater than 100 μM are toxic for microbial cultures might be the reasons why it has been difficult to isolate pure cultures of microorganisms able to grow on tetralin as the only carbon and energy source [3]. Tetralin utilization by microorganisms has long been reported by mixed cultures or by pure cultures through co-oxidation of tetralin in the presence of mixed substrates [5]. Reports in the literature describing poor growth on tetralin with pure cultures are from *Pseudomonas stutzeri* AS39 [6] and *Corynebacterium* sp. strain C125 [7]. By identifying accumulated intermediates, it was suggested that they might attack the molecule of tetralin in different ways. In one, the alicyclic ring is initially hydroxylated and then oxidized [6] whereas in the other the aromatic ring is dioxygenated and subsequently cleaved in the extradiol position [7]. The degradation pathway has not been further studied in any of these strains. The main source of knowledge about tetralin degradation has been provided *by Sphingopyxis granuli* strain TFA (formerly *Sphingomonas macrogolitabida* strain TFA) where the catabolic pathway has been completely characterized at biochemical, genetic and regulatory levels. A similar degradation pathway has been described in *Rhodococcus* sp. strain TFB based on the induction in tetralin-grown cells of homologous enzymes to the TFA ones. These two different bacteria were isolated from the river Rhine sediments after selection for growth on tetralin. TFA is a Gram-negative α-proteobacterium, whose genome is available and shows characteristics of oligotrophic bacteria, great plasticity and presence of other degradative genes that might confer new capabilities to this bacterium [8]. Moreover, TFA is the first of its genus, generally described as aerobic, in which the ability to grow in the absence of oxygen using nitrate as a terminal electron acceptor has been demonstrated [8]. This capacity might allow TFA to invade other niches. TFB is a Gram-positive Actinobacterium from the *Nocardiaceae* family refractory to genetic manipulation, although a proteomic approach has allowed the identification of all the tetralin gene clusters. In this review, we summarize the genetic organization, the biochemistry of the tetralin biodegradation pathway and its connection with the central metabolism via production of pyruvate and acetil-CoA in both strains. We also highlight the contributions that the study of the tetralin degradation genes regulation in each strain has made to understand the regulation of gene expression of other degradation pathways, and to the knowledge of bacterial mechanisms of gene regulation in general.

## 2. Identification and Organization of Tetralin Degradation Gene Clusters 

As mentioned above, the tetralin biodegradation pathway has been elucidated only in two bacteria isolated from the same environmental niche, *Sphingophyxis granuli* strain TFA and *Rhodococcus* sp. strain TFB. In this section, we review the different methodology employed to identify *thn* genes and the organization of the *thn* gene clusters in both strains. 

### 2.1. Organization of the Gene Clusters in TFA

**Figure 1:**
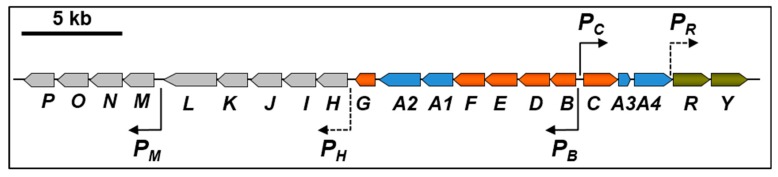
Organization of the chromosomal *thn* genes in TFA. The arrows show the directions of gene transcription. Bent arrows represent the identified promoters. Dashed lines represent internal promoters.

A Tn5 or miniTn5*Km* transposon mutant isolation approach was employed to identify *thn* genes in TFA. A gene bank of TFA was constructed [9] and used to complement transposon insertion mutants. Cosmids containing genes that complemented the growth deficiency on tetralin of transposon mutants were obtained and sequenced ([10] and references therein). As shown in Figure 1, the regulatory and structural *thn* genes consist of 21 genes comprised in a 14.3-kb DNA fragment and organized in four closely linked operons (B, C, H and M). The divergent operons C and B have two internal promoters *P_R_* and *P_H._*

Operon C contains five genes: *thnC*, encoding an extradiol dioxygenase, *thnA3* and *thnA4*, coding for the ferredoxin and the ferredoxin reductase components of the complex catalysing the initial dioxygenase reaction, and the regulatory genes *thnRY*. ThnR belongs to the LysR-type transcriptional regulators (LTTR) family. In most cases, the gene encoding the LTTR is divergently transcribed to the regulated operon [11]. Thus, *thn* genes have an unusual organisation in TFA being that the regulatory genes are part of a structural operon while also being transcribed from the internal and constitutive promoter *P_R_*. In turn, *thnY* codes for a ferredoxin reductase-like protein, which has a transcriptional regulatory function. This is unusual for an electron transport protein, thus representing another particularity of the tetralin biodegradation genes of TFA (see below). 

Operon B comprises 12 genes: *thnB*, which encodes a *cis*-dihydrodiol dehydrogenase, *thnD*, coding for the key hydroxylase that renders a linear compound, *thnE,* encoding a hydratase, *thnF,* encoding an aldolase, *thnA1* and *thnA2*, encoding the α- and β-subunits of the initial dioxygenase, respectively, and *thnG*, coding for a non-acylating aldehyde dehydrogenase. Five additional genes (*thnHIJKL*) are comprised within operon B although also transcribed from the internal promoter *P_H_*. These genes encode proteins similar to those involved in β-oxidation pathways. 

Finally, operon M comprises *thnM*, encoding a TonB-dependent protein of unknown function, *thnN*, encoding a putative glutaryl-CoA dehydrogenase and *thnO* and *thnP* which encode proteins similar to the α- and β-subunits of a flavoprotein, respectively [10].

A few years ago, the complete genome sequence of TFA was obtained and analysed [8]. The presence of large plasmids in strains of the sphingomonas group able to degrade different contaminants is very common. In fact, a number of “degradative megaplasmids” have been described in this group of bacteria that contribute to their catabolic flexibility. Some examples are pNL2, pCAR3, pSWIT02, pCHQ1, pISP0, and pISP1, which code for genes involved in the degradation of aromatic hydrocarbons, carbazole, dibenzo-p-dioxin and γ-hexachlorocyclohexane, respectively [12]. In contrast, no plasmids were detected in TFA by experimental approaches, which was later confirmed when the genome was sequenced. The *thn* genes were found within a chromosomal region identified as a putative genomic island and relatively close to a *tra*/*trb* gene cluster. Although more data are needed to be conclusive, those regions could be considered as integrative and conjugative elements (ICEs) [13] which might have played an important role in the final structure of the TFA genome. The analysis performed in 2016 to define the core genome of the *Sphingopyxis* genus showed that all tetralin degradation proteins, except ThnA4, ThnY and ThnM, are part of this core genome [8].

### 2.2. Organization of the Gene Clusters in TFB

**Figure 2:**
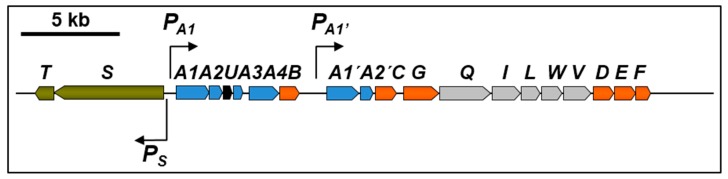
Organization of the chromosomal *thn* genes in TFB. The arrows show the directions of gene transcription. Bent arrows represent the identified promoters.

TFB is a bacterium refractory to genetic manipulation and *thn* gene identification had to be carried out by reverse genetics using a proteomic approach [14]. This method has also been used to identify biodegradation genes in other bacteria of environmental interest [15]. Soluble proteins present in extracts of TFB cells grown on glucose or on tetralin were compared using 2D-DIGE (two-dimensional difference gel electrophoresis). Differentially expressed proteins present in tetralin cell extracts were identified by MALDI-MS(MS) (maldi-assisted laser desorption/ionization tandem mass spectrometry) or ESI-IT MS/MS (electrospray ionization-ion trap tandem mass spectrometry). Eight proteins with probable roles in the conversion of tetralin into a linear compound were identified, ThnD being one of them. Degenerated primers based on the ThnD-obtained peptide sequences were designed to amplify a DNA fragment of the putative *thnD* gene, which was used to screen a TFB cosmid library. A DNA fragment comprising 18 putative *thn* structural genes and two more coding for the putative two-component regulatory system *thnST* were sequenced. *thnS* encodes for a membrane sensor protein and ThnT is similar to members of the LuxR family of regulators. As shown in Figure 2, TFB *thn* structural genes are organized into two operons, *thnA1A2A3UA4B* and *thnA1’A2’CGQILWVDEF*, transcribed from *P_A1_* and *P_A1’_* promoters, whereas the regulatory operon is divergently transcribed to the structural ones from the *Ps* promoter.

All genes encoding enzymes are similar to those found in TFA except *thnU*, coding for a putative sterol transfer protein, *thnQ*, encoding a pimeloyl-CoA synthase, *thnW* coding for a 3-hydroxyacyl-CoA dehydrogenase and *thnV* coding for an acyl-CoA dehydrogenase (see Table 1). TFB *thn* genes are highly similar to *bph* and *etb* genes in *R. jostii* sp RHA1, which are located on pRHL1 and pRHL2 plasmids. Similarly, using pulse field electrophoresis analysis, two large plasmids, pTFB1 and pTFB2, with estimated sizes of 1100 and 280 kb, respectively, have been identified in TFB, being the *thn* genes located on pTFB1 [14].

## 3. Tetralin Degradation Pathway and Enzymes Involved

**Scheme 1:**
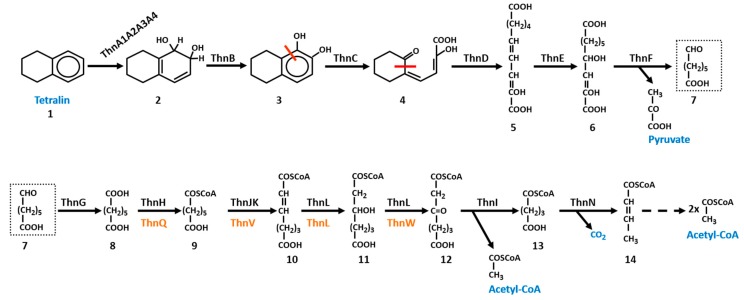
Tetralin catabolic pathway. Compound designations: **1**, 1,2,3,4-tetrahydronaphthalene (Tetralin); **2**, 1,2-dihydroxy-1,2,5,6,7,8-hexa-hydronaphthalene; **3**, 1,2-dihydroxytetralin (DHT); **4**, 4-(2-oxocyclohexyl)-2-hydroxybuta-2,4-dienoic acid (OCHBDA); **5**, 2-hydroxydeca-2,4-dienedioic acid; **6**, 2,4-dihydroxydec-2-ene-1,10-dioic acid (DHDDA); **7**, 7-Oxoheptanoic acid (pimelic semialdehyde); **8**, heptanedioic acid (pimelic acid); **9**, pimeloyl-CoA; **10**, 2,3-dehydropimeloyl-CoA; **11**, 3-hydroxypimeloyl-CoA; **12**, 3-oxopimeloyl-CoA; **13**, glutaryl-CoA; **14**, crotonyl-CoA. Proteins in brown correspond to those identified in TFB. Red lines show the cleavage of the rings.

In this section, the main features of the tetralin degradation pathway in TFA and TFB are reviewed. Although the degradation pathway is essentially the same in both bacteria (Scheme 1), differences have been found in some of the enzymes. The proteins involved in the degradation pathway to convert tetralin into pimelic acid were quite similar in both bacteria except for ThnA4 (Table 1). This similarity ranges from 65% of identity for ThnC proteins to 36% for ThnG enzymes. In all cases, coverage is higher than 90%. The main difference between TFA and TFB pathways was found in those proteins involved in pimelic acid conversion to central metabolites via a β-oxidation pathway [14,16]. It should be noted that the tetralin degradation pathway has been characterised in detail only in strain TFA. The existence of similar enzymes led to the conclusion that a similar pathway should operate in TFB.

Aerobic biodegradation of tetralin starts by a common mechanism quite well described for degradation of aromatic compounds, which is the insertion of oxygen into the aromatic ring of tetralin by a non-heme iron-containing dioxygenase [17]. These multicomponent ring-hydroxylating enzymes require Fe^2+^ and an electron supply in addition to molecular oxygen. These enzymes establish an electron-transport chain that allows the transfer of electrons from NAD(P)H, via flavin and [2Fe-2S] redox centers, to the terminal oxygenase containing the catalytic site. Bacterial oxygenases has been classified into classes I, II, and III according to the number of components and the nature of the redox centers of the electron transport chain [18]. Class I consists of two components, the reductase and the oxygenase. The reductase contains a chloroplast type [2Fe-2S] cluster and a flavin, FMN in class IA and FAD in class IB. Class II consists of three-component enzymes but in this case, the flavin and [2Fe-2S] redox centers of the reductase are on a separate flavoprotein and ferredoxin, respectively. Class III consists of three-component enzymes in which the reductase harbours both FAD and a chloroplast type [2Fe-2S] cluster and transfer electrons to an intermediary Rieske type ferredoxin rather than directly to the oxygenase component [18]. The tetralin dioxygenase system belongs to class III, composed of the dioxygenase complex itself, formed by ThnA1 and ThnA2, the α and β subunits of the initial tetralin dioxygenase, respectively, and two electron transfer components, the ferredoxin ThnA3 and the ferredoxin reductase ThnA4. Both ThnA1^TFA^ and ThnA1^TFB^ amino acid sequences contain two Cys and two His residues, (CXHX_15–17_CX_2_H), that co-ordinate the Rieske-type [2Fe-2S] cluster and the other catalytic residues that coordinate the mononuclear iron, identified in other crystallized dioxygenases, such as the one from *Pseudomonas* sp. NCIB 9816–4 [19]. ThnA3 is homologous to the Rieske-type ferredoxin components that are electron transfer intermediates between the NAD(P)H-dependent ferredoxin reductases and the dioxygenase components. ThnA4^TFA^ TFA is homologous to the class III NAD(P)H-ferredoxin reductases of degradative pathways. The modular structure of these ferredoxin reductases is composed of a N-terminal domain that contains a conserved Cys-X_4_-Cys-X_2_-Cys-X_29/30_-Cys motif that binds a plant-type [2Fe-2S] cluster, and a central and C-terminal domains with the conserved motifs for flavin and NAD(P)H binding, respectively [20]. However, ThnA4^TFB^ is 81.5% identical to EtbAd from *Rhodococcus jostii* RHA1, a protein with Pyr_redox_2 domains which are present in pyridine nucleotide-disulphide oxdoreductases, a family that includes both class I and class II oxidoreductases.

Both ThnA4^TFA^ and ThnA3^TFA^ have been purified, their redox centres spectroscopically confirmed, the specific sequence in the electron transfer reaction from ThnA4_red_ to ThnA3_ox_ has been analysed using stopped-flow spectroscopy, and their midpoint reduction potentials have been determined. These results agree with the electron transfer from NADPH to FAD and then to [2Fe-2S]) in ThnA4, and subsequently to [2Fe-2S] cluster in ThnA3 [21]. Interestingly, this transfer of electrons is important not only for the initial oxidation of tetralin but also for the correct regulation of the *thn* genes expression (see Section 4), since a new electron transfer route from ThnA4 and ThnA3 to the regulatory protein ThnY may control the activity of the regulators of *thn* genes in response to particular substrates. The absence of ThnY in TFB and the different regulatory system for *thn* gene expression in this bacterium might explain the lack of ThnA4 conservation between TFA and TFB. Experimental evidence that ThnA1A2A3A4^TFA^ are directly involved in the initial dioxygenation of tetralin is provided by accumulation of 2HT (2-hydroxy-5,6,7,8-tetrahydronaphthalene), which could be determined by HPLC, in the supernatant of a TFA mutant lacking all *thn* genes supplied with tetralin and overexpressing ThnA1A2A3 [17]. Although ThnA4 is not essential for 2HT production, maximal production of 2HT is achieved only in its presence. According with this, mutants in ThnA1, ThnA2, or ThnA3 are unable to grow on tetralin. ThnA4 is partially dispensable and its function is apparently substituted by other ferredoxin reductases present in TFA, as it has been described for other ring-hydroxylating dioxygenases [18].

The next reaction of the catabolic pathway is catalysed by the *thnB* gene product. ThnB is a *cis*-dihydrodiol dehydrogenase highly homologous to those involved in phenanthrene degradation. A ThnB^TFA^ mutant is unable to grow on tetralin. However, when this mutant is grown in minimal medium containing 8 mM β-hydroxybutyrate (βHB) and tetralin as carbon sources, a condition that allows growth of Thn-mutants while expressing the *thn* genes, a mixture of 1HT (1-hydroxy-5,6,7,8-217 tetrahydronaphthalene) and 2HT (2-hydroxy-5,6,7,8-tetrahydronaphthalene) were detected by high-performance liquid chromatography (HPLC) in the supernatant of the growth culture after acidification. Formation of 1HT and 2HT indicated that the metabolite accumulated in ThnB mutant corresponds to *cis*-dihydrodiol 1,2-dihydroxy-1,2,5,6,7,8-hexahydronaphthalene (1,2-DHT) since dihydrodiols are unstable under acidic conditions and this results in the formation of rearomatized monohydroxylated derivatives [17].

ThnC is the extradiol dioxygenase required for tetralin utilization. Extradiol dioxygenases are key enzymes in the aerobic degradation of many aromatics compounds. They function by cleaving the meta-position of the C–C bond of catechol derivatives, leading to the aromatic ring opening with the concomitant incorporation of two oxygen atoms [22]. ThnC^TFA^ has been purified, and biochemically characterized. The estimated molecular mass of ThnC indicated that it is a decamer [23], which is different from the octameric structures reported for other extradiol dioxygenases closely related to ThnC, such as BphC [24], NahC [25], and EtbC [26]. TFA *thnC* mutants are unable to grow on tetralin and accumulate 1,2-DHT in the supernatant when growing on 8 mM βHB and tetralin [23]. The product of the reaction catalysed by ThnC is 4-(2-oxocyclohexyl)-2-hydroxy-buta-2,4-dienoic acid (OCHBDA), a yellow ring product resulting from the cleavage proximal to the alicyclic ring of 1,2-DHT that exhibits the typical behaviour of a hydroxymuconic semialdehyde. In addition to 1,2-DHT, the range of substrates of ThnC is relatively wide. It showed activity towards 1,2-dihydroxynaphthalene (1,2-DHN), the intermediate in the naphthalene biodegradation pathway, and to a lesser extent, towards 2,3-dihydroxybiphenyl (2,3-DHB), the intermediate in the biphenyl biodegradation pathways, and the methylated catechols, 3-methylcatechol or 4-methylcatechol [23,27]. This broad substrate specificity of ThnC is also shown by other 1,2-dihydroxynaphthalene dioxygenases [25]. ThnC and these enzymes cluster together in group 2 of the I.3.E subfamily of two-domain extradiol dioxygenases, which preferentially cleave bicyclic substrates [23,28]. As shown by a metagenomic survey, dioxygenases of the I.3.E subfamily are the ones with the broadest substrate specificity [29]. Mutational analysis of ThnC^TFA^ showed that it is possible to obtain altered ThnC proteins with modified positions surrounding the substrate-binding site (Q198H, G206M, N213H, A282R and A282G) that improved activity and/or specificity for 1,2-DHT, thus indicating that ThnC activity is not maximized for this substrate.

Hydrolases of *meta*-cleavage pathways are a class of β-ketolases which catalyse the hydrolysis of linear C-C bonds of vinylogous 1,5-diketones formed by the dioxygenative *meta*-ring cleavage of arenes. Most of them belong to the *α/β*-hydrolase-fold superfamily of enzymes. These enzymes are structurally, functionally and mechanistically related and generally utilize a nucleophilic residue (a serine in most cases)-acidic residue-histidine catalytic triad to hydrolyse the C-C bond adjacent to a carbonyl [30]. The serine hydrolase ThnD from TFA has been purified and biochemically characterized [31]. ThnD^TFA^ is an octameric enzyme with an optimum reaction temperature at 65 °C. Moreover, it shows high stability at room temperature and heat treatment at 70 °C for 2 h resulted in 78% of the initial activity. ThnD^TFB^ has also been heterologously expressed in *Escherichia coli* and purified, and its optimal temperature is 70 °C [14]. This stability at high temperature is a biotechnological interesting property given the use of this class of enzymes in organic synthesis [32]. ThnD^TFA^ and ThnD^TFB^ catalyse the hydrolysis of OCHBDA rendering the dienol 2-hydroxydec-2,4-diene-1,10-dioicacid as the reaction product. In fact, a TFA insertion mutant defective in ThnD accumulates OCHBDA [31]. It has been suggested that ThnD could be involved in biphenyl meta-cleavage pathways since it is highly similar to BphD enzymes and, in addition to OCHBDA, ThnD also efficiently hydrolyses 2-hydroxy-6-oxo-6-phenylhexa-2,4-dienoic acid (6-phenyl-HODA), the ring fission product of the biphenyl meta-cleavage pathway. In fact, the *K_m_* for OCHBDA and for 6-phenyl-HODA are 26.3 mM and 34.8 mM, respectively [31]. ThnD^TFB^ hydrolase is the most similar to BphD hydrolases and even more active towards 6-phenyl-HODA than towards OCHBDA, which showed just 40% of the activity obtained with 6-phenyl-HODA [14]. ThnD^TFA^ does not hydrolyse 6-methyl-HODA, aldehydes HODA or 5-methyl-HODA, ring fission products of 3-methylcatechol, catechol and 4-methylcatechol, equivalent intermediates of *meta*-cleavage pathways of mono-aromatic compounds. These compounds have in common the small substituents in C-6. Interestingly, since the bond of the vinylogous β-diketone connecting C-5 and C-6 in OCHBDA is within the alicyclic ring of tetralin derivative, hydrolysis of this bond by ThnD renders a single linear dicarboxylate product instead of the two products usually generated by these hydrolases (Scheme 1). Concomitantly, this hydrolysis breaks open the recalcitrant alicyclic ring, thus rendering a more easily catabolised product. This confers unique properties to the tetralin degradation pathway, since ThnD makes unnecessary the recruitment of enzymatic activities involved in degradation of alicyclic rings for tetralin complete mineralization.

Both ThnE and ThnF showed similarities with the corresponding hydratases and aldolases involved in the catabolic degradation of homo-protocatechuate, and their substrates and products of the reactions they catalyse have been identified [33]. GC-MS analysis of the reaction products using OCHBDA as a substrate and crude extracts of an *E. coli* strain overproducing ThnD and ThnE, identified a C10 dicarboxylic compound with two hydroxyl groups, the enol tautomer 2,4-dihydroxydec-2-ene-1,10-dioic acid (DHDDA) as the major product resulting from the hydration reaction [33]. Although it has been suggested that both the dienol and the keto tautomers of the ThnD product could be substrates of ThnE, in TFA the enol form is the most probable since when purified hydrolase ThnD^TFA^ was used to transform OCHBDA, only the dienol tautomer reaction product was detected [31].

ThnF is a class II aldolase encoded by the *thnF* gene, whose start codon in TFA is just six nucleotides downstream of the stop codon of *thnE* gene [33]. A similar situation is found in TFB where *thnF* start codon is within the *thnE* coding sequence [14]. This indicates that translational coupling is highly probable for these genes. Aldolases are an evolutionarily diverse class of enzymes that are typically divided into two groups based on their catalytic mechanism. The Class I aldolases utilize a Schiff base mechanism to stabilize a carbanion intermediate, whereas the Class II enzymes utilize divalent metal ions to stabilize an enolate intermediate. Within each mechanistic class of aldolases, further diversity exists with respect to the sequence, structure, and substrate specificities. Pyruvate-specific enzymes are within the Class II containing an octahedral coordinated divalent metal cofactor, which is usually Mg^2+^, Mn^2+^ or Co^2+^ [34]. Transformation of OCHBDA by *E. coli* extracts of enriched in ThnD, ThnE, and ThnF enzymes from TFA resulted in accumulation of two compounds as a result of the cleavage reaction, pimelic semialdehyde, identified by GC-MS, and pyruvate, which was spectrophotometrically estimated [33]. 

For complete oxidation, pimelic semialdehyde needs to be further metabolized into compounds that can enter the Krebs cycle. A first oxidation of pimelic semialdehyde leading to the synthesis of pimelic acid is enzymatically carried out by ThnG, which is similar to the non-acylating NAD-dependent aldehyde dehydrogenases involved in the biodegradation pathways of aromatic compounds. Aldehyde dehydrogenases (ALDHs) catalyse the oxidation of aldehydes to their corresponding carboxylic acids and all of them require either NAD or NADP as a cofactor [35]. The catalytic activity of ThnG has been assayed in crude extract of TFA and in a ThnG^TFA^ *E. coli* overproducing strain [10]. ThnG shows ALDH activity depending on pimelic semialdehyde and NAD as an oxidant but not with NADP, and its activity does not show coenzyme A or dithiothreitol dependence, supporting the non-acylating ALDH activity of ThnG. Indeed, the primary structure of ThnG^TFA^ contains the four invariant residues (Gly228, Gly281, Glu381, and Phe383) found in this type of dehydrogenases, which are critical for binding of the nicotinamide ring of NAD and catalytic activity. These residues are also present in ThnG^TFB^ in similar locations. The participation of ThnG in the catabolism of tetralin is clear since ThnG activity and *thnG* gene expression as part of the B operon is induced in TFA cells growing on tetralin. However, as it happens in *thnA4* mutants, TFA *thnG* mutants are able to grow on tetralin. Therefore, it seems that this role can be assumed by other aldehyde dehydrogenases present in TFA that could also use pimelic semialdehyde as a substrate [10]. Since ThnG is not an acylating enzyme, the formation of pimeloyl-CoA to enter a β-oxidation pathway needs an intermediate step consisting in the activation of the pimelic acid molecule. 

Based on the homology of the *thnHIJKL* gene product with enzymes that participate in β-oxidation pathways, a complete β-oxidation pathway for pimelic acid has been described in TFA. The steps involved are: initially, ThnH, a protein similar to enzymes that belong to the “CaiB/BaiE” family of CoA transferases that catalyse the reversible transfer of CoA groups from CoA thioesters to free acids, would activate pimelic acid yielding pimeloyl-CoA. In TFB, the same step seems to be catalysed by ThnQ, a protein similar to pimeloyl-CoA synthases that catalyses the binding of a CoA-SH molecule to pimelic acid. In TFA, the resulting pimeloyl-CoA molecule would be the substrate of ThnJK, which encodes proteins similar to the large and small subunits of a pimeloyl-CoA dehydrogenase, respectively. This enzyme might oxidise pimeloyl-CoA to pimeloyl-2,3-dehydroacyl-CoA acid. In TFB, ThnV could be the acyl-CoA dehydrogenase responsible of catalysing this step. In TFA, a bifunctional enzyme (ThnL) carrying the enoyl-CoA hydratase and 3-hydroxyacyl-CoA dehydrogenase domains located at its N and C termini, respectively, could convert the pimeloyl-2,3-dehydroacyl-CoA acid into pimeloyl-3-ketoacyl-CoA in two steps. In TFB, ThnL would render pimeloyl-2,3-hydroxyacyl-CoA, which would be oxidised to the ketoacyl-CoA by ThnW. Finally, in both bacteria, ThnI, encoding an acetyl-CoA acetyl transferase, would cleave pimeloyl-3-ketoacyl-CoA into the central metabolites acetyl-CoA and glutaryl-CoA. In TFA, ThnN, encoding a putative glutaryl-CoA dehydrogenase, could catalyse the oxidative decarboxylation of glutaryl-CoA to crotonyl-CoA, which could be further converted into acetyl-CoA. Moreover, two extra proteins, ThnO and ThnP encode proteins similar respectively to the α- and β-subunits of flavoproteins, which serve as specific electron acceptors for primary dehydrogenases of the β-oxidation pathway and transfer electrons to the respiratory chain. Proteins similar to ThnN, ThnO and ThnP have not been found associated to *thn* genes in TFB. Mutations in *thnH*, *thnI*, *thnJ* or *thnK*, did not affect the growth of TFA on tetralin, or on sebacic or pimelic acids [10]. Thus, redundant β-oxidation pathways should be present in TFA cells. Actually, there are 39 proteins annotated in TFA genome within the GO terms GO:0006635 (fatty acid beta-oxidation) and GO:009062 (fatty acid catabolic process) [8].

Taking all the steps together, degradation of one molecule of tetralin by this pathway renders one molecule of pyruvate, one of acetyl-CoA and one of glutaryl-CoA (Scheme 1). These molecules can be used either as energy or carbon source. The use of the same degradation strategy by two non-phylogenetically related bacteria and different gene arrangements could mean that it is the most efficient pathway to obtain carbon and energy source from tetralin.

Interestingly, *thn* genes are also induced by naphthalene in both TFA and TFB [36,37]. However, and despite the ThnC activity towards 1,2 DHN, TFA is unable to grow on naphthalene while TFB can use it as the only carbon and energy source [37]. In fact, Thn proteins are the most prominent in extracts of naphthalene-grown TFB cells. Because of that, it has been proposed that ThnA1A2A3A4, ThnB and ThnC proteins also participate in the naphthalene degradation pathway in TFB and that the TFB naphthalene degradation ability actually results from the combination of different catabolic pathways [37].

## 4. Regulation of the *thn* Gene Expression

In this section we review the regulatory elements and their function controlling the expression of the tetralin degradation genes in TFA and TFB. In both strains, tetralin degradation genes are subjected to a specific induction by tetralin but also to an over-imposed global control, which dictates the use of other preferential carbon sources when present. The regulatory mechanisms behind these responses are summarized.

### 4.1. Specific Regulation: Elements Involved in Tetralin Induction 

#### 4.1.1. Tetralin Induction in TFA

When TFA cells are growing on tetralin as the sole source of carbon and energy, the expression of the *thn* genes from *P_C_* and *P_B_* promoters is induced more than 100-fold compared to when growing on the preferential carbon source βHB [38]. In many degradative routes, induction of the pathway requires an intermediate in the catabolism of the substrate [39,40,41]. However, induction of *thn* genes is exerted directly in response to tetralin, the substrate of the catabolic pathway, since some mutants unable to transform it are still able to activate *thn* gene transcription [36]. Three regulatory proteins achieve this specific response to tetralin. ThnR, a LysR-type regulator involved in the recognition of potential effector molecules, ThnY, the ferredoxin like reductase that has evolved to function as a ThnR co-activator, both essential for *thn* gene expression, and ThnA3, the ferredoxin that delivers electrons to the tetralin dioxygenase and that prevents promiscuous induction by molecules that are not substrates of the catabolic pathway [38]. Details of each regulatory element are described below. 

ThnR has exclusive properties. Firstly, unlike other LysR-type regulators, it does not repress its own synthesis, but activates its own expression in a positive circuit of regulation, by being part of the same transcriptional unit as the catabolic genes *thnCA3A4* located upstream (Figure 1) [38]. It has been proposed that these positive auto-regulation circuits may allow a faster switch on/off of the regulatory systems in response to the inducer molecules, for example, in environments where the amount of substrate is limited [42]. This might be the case for tetralin considering its tendency to accumulate into biological membranes [4]. Secondly, ThnR needs the regulatory protein ThnY for transcription activation [17]. In this way, ThnR is one of the few examples of LysR-type activator that requires an additional protein to activate transcription [43]. Additionally, ThnY allows the system to integrate other signals, such us the presence of potential inducers that, being similar to tetralin, are not substrates of the pathway and thus preventing a wasteful production of useless catabolic enzymes (see ThnA3 modulation below). 

The molecular mechanism responsible for *thn* gene induction in the presence of tetralin has been characterized. According to it, basal levels of ThnR and ThnY are provided by the weak constitutive promoter *P_R_*, which in turn are responsible for the activation of the four promoters, *P_B_*, *P_C_*, *P_H_* and *P_M_*, when tetralin is present [10]. Footprinting analyses have shown that ThnR binds to contiguous high affinity and low affinity sites in each promoter region [16]. A meticulous analysis of ThnR binding to the *thnB*–*thnC* intergenic region, (*P_B_*–*P_C_* promoters), led to a detailed molecular characterization of the activation mechanism mediated by ThnR and the importance that both primary and secondary binding sites have on transcription activation from each promoter. These results showed that mutations in the primary binding sites of each promoter can affect not only the transcriptional activation of its respective promoter but transcription from the other one, and vice versa. This transcriptional coordination is mediated by a complex structure of ThnR molecules that interact among them when bound to their respective sites in both promoters via a DNA loop, which is detected as a higher order structure in electrophoretic mobility shift assays (EMSA) [10]. For this DNA loop to be formed, primary binding sites of each promoter have to be aligned in the same face of the helix, since insertions of 4 and 6 bp between them prevented the formation of the complex structure and cooperative transcription activation. Furthermore, the DNA loop functions by compensating non-productive ThnR-DNA interactions at an activating site [16,44].

Similarly to ThnR, *P_R_* also provides basal levels of ThnY, which are increased from the inducible *P_C_* promoter in a ThnR-dependent manner when tetralin is present [45]. As mentioned above, ThnY is essential for expression of each of the four *thn* promoters controlled by ThnR. ThnY is homologous to the Class III of ferredoxin reductases of degradation of aromatic compounds [18], such as benzoate 1,2-dioxygenase reductase [46], naphthalene dioxygenase reductase [47] and carbazole dioxygenase reductase [48]. These ferredoxin reductases function by accepting electrons from NAD(P)H and transferring them through the FAD and [2Fe-2S] cluster, to an intermediary Rieske-type ferredoxin, which finally transfer the electrons to the dioxygenase. Purified ThnY contains a non-covalently attached FAD and one plant-type [2Fe-2S] cluster characteristic of this class of ferredoxin reductases [45]. Regarding these features, ThnY is similar to the ferredoxin reductase ThnA4 of the tetralin dioxygenase [21]. However, despite having these redox centers, ThnY does not behave like a NAD(P)H-dependent ferredoxin reductase, since neither can be reduced directly by NAD(P)H nor does it have the enzymatic activities of these enzymes, such as diaphorase and/or cytochrome *c* reductase activity. Actually, ThnY lacks a proper NAD(P)H binding in its C-terminal domain [45], such as the conserved glycine signature (GGXGXXP) proposed to be involved in the binding of the adenosine-5-phosphate groups of NADH or 2´-phospho-AMP of NADPH [20]. Knowing why ThnY is necessary for ThnR-mediated transcription activation has been one of the most interesting findings on *thn* genes regulation. Footprint and DNA binding assays revealed that when oxidised, ThnY does not alter the footprint pattern generated by ThnR but it does increase the affinity of ThnR for the *P_C_* promoter region and stabilize the structure of the protein-DNA complex, as a new defined complex is detected in its presence in EMSA assays. It has been proposed that ThnY co-activates transcription by interacting with ThnR [45]. This regulatory function of ThnY is of remarkable interest since it is the first example of a ferredoxin like reductase of the dioxygenases systems that works as an additional sensor regulating gene expression [45,49]. 

The role of ThnY as a new redox sensor linking cellular metabolism to gene expression has also come from the experiments carried out with the ferredoxin ThnA3 [16,21,50]. As mentioned above in Section 3, ThnA3 is a Rieske-type ferredoxin that functions by transferring electrons to the tetralin dioxygenase, but in addition to this catalytic function, ThnA3 has a regulatory role as a discriminator. There are several lines of evidences that show that ThnA3 reports to ThnY whether a molecule is a good or a bad substrate of the tetralin dioxygenase through its redox state and that this communication is established by modifying the redox state of ThnY. Firstly, ThnA3 has a discriminatory role since in *thnA3* mutants, when only ThnR and ThnY are present, the *thn* promoters are activated promiscuously, producing high levels of wasteful expression with effector molecules that are not substrates of the first step of the tetralin catabolic pathway, such as *cis*-decalin, cyclohexane, *trans*-decalin, or benzene [16]. This phenomenon of gratuitous induction has also been described in other degradative pathways [51]. Secondly, reduced ThnA3 has a negative effect on *thn* expression since mutants strains lacking either the α (ThnA1) or β (ThnA2) subunits of the dioxygenase, a condition resulting in an accumulation of the reduced form of ThnA3, failed to induce properly the expression of *thn* genes even in the presence of the substrate tetralin [16]. Under this physiological condition, reduced ThnA3 is fully available to interact with ThnY. Thirdly, ThnA3 is the electron partner of ThnY. ThnY variants, ThnY-C40S and ThnY-N201G,S206P, affected in the iron sulfur cluster and in the degenerated NAD(P)H binding domain, respectively, render ThnA3 useless for signal transduction as the discrimination capacity is completely lost and the expression of *thn* genes occurs at high levels even in the presence of molecules other than tetralin. Thus, the discriminatory function of ThnA3 is exerted through the redox protein ThnY [50]. By using stopped-flow spectrophotometry and determination of midpoint reduction potentials of each redox cofactor within ThnA4, ThnA3 and ThnY, it has been possible to show that ThnY can actually be reduced by ThnA3, according to the following ordered steps during the electron transfer reactions [21]:

NADH → ThnA4FAD → ThnA4[2Fe-2S] → ThnA3[2Fe-2S] → ThnYFAD → ThnY[2Fe-2S].

**Figure 3:**
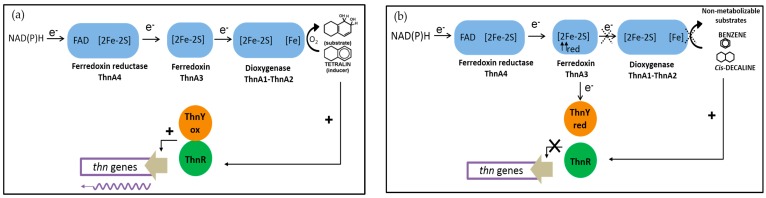
Model for specific regulation and modulation of *thn* genes transcription in TFA. (**a**) In the presence of the tetralin, electrons are preferentially delivered to the dioxygenase and ThnY_ox_ and ThnR can activate transcription from the inducible *thn* promoters. (**b**) In the presence of compounds that are non-substrates of the dioxygenase, ThnA3 is accumulated in the reduced state and electrons are redirected towards ThnY instead of the dioxygenase impairing *thn* genes transcription.

Purified ThnA4 behaves as a typical ferredoxin reductase of dioxygenase systems, able to be reduced preferentially by NADH, able to reduce the ferredoxin ThnA3, and with midpoint potentials for *E*_ThnA4FADox/hq_ and *E*_ThnA4SFeox/red_ of −200 mV to −150 mV ranges respectively [21], similar to other reported NAD(P)H ferredoxin reductases of the dioxygenase systems [52]. Similarly, ThnA3 behaves like the one electron carrier Rieske-type [2Fe-2S] ferredoxin although with an *E*_ThnA3SFeox/red_ (−112 ± 5 mV) [21] less negative than reported ferredoxin [53]. *E*_ThnYFADox/hq_ and *E*_ThnYSFeox/redwith_ with values of −131 ± 8 mV and −136 ± 8 mV, respectively, are less negative too than those of other ferredoxin reductases counterparts and slightly more negative than that of ThnA3. However, ThnY can only be reduced by ThnA3_red_ but not by NAD(P)H. The redox potentials also highlight that in order to reduce ThnY, the reduced form of ThnA3 has to be predominant, thus allowing the equilibrium of the redox reaction to change toward ThnY [21]. Figure 3 shows the regulatory model for *thn* genes expression which implies two electron transport routes: (i) NAD(P)H-ThnA4–ThnA3–ThnA1/ThnA2 that occurs in the presence of tetralin, in which tetralin dioxygenase acts as an electron sink through the dioxygenation reaction. Reduction of ThnY by ThnA3 is minimal thus allowing ThnR and ThnY_ox_ to activate the *thn* promoters and (ii) the regulatory NAD(P)H–ThnA4–ThnA3–ThnY that occurs in the presence of non-efficient substrates of the dioxygenase, thus leaving an abundant ThnA3_red_ form fully available to interact with ThnY and to impair *thn* expression. This represents an unprecedented regulatory system in which components of an electron transfer system of a catabolic pathway prevent its gratuitous induction by molecules that cannot be effectively metabolized.

#### 4.1.2. Tetralin Induction in TFB

Semiquantitative RT-qPCR analysis of *thn* genes expression in TFB showed the induction of structural and regulatory genes in tetralin-grown cells in three operons (Figure 2). In glucose- or glucose plus tetralin-grown cells, regulatory genes were expressed at low level while transcription of structural genes was undetected in glucose-grown cells and partially repressed in glucose plus tetralin-grown cells [14]. Primer extension experiments allowed to establish the transcription start points for *thnS* and *thnA1* (which is the same as *thnA1’*) and the putative sequences of the divergent promoters [54]. An 18 bp sequence, similar to those present in biphenyl-inducible promoters, was found in *thnA1* and *thnA1’* promoters. Point mutations in this sequence negatively affected tetralin induction of a *thnA1::gfp* translational fusion indicating that those nucleotides could be recognised by a transcriptional activator. Moreover, introduction of extra copies of *thnST* into TFB resulted in higher expression of *thnA1::gfp* in all conditions. Taken together, those results suggested that the membrane sensor ThnS recognises a signal in the presence of tetralin, and that, in response, the regulator ThnT binds the promoters to induce *thn* gene expression in TFB [54].

### 4.2. Carbon Catabolite Repression 

One of the major drawbacks of microbial degradation of pollutant compounds is the repression of the catabolic genes in the environment due to the presence of preferential carbon and energy sources. However, from an energetic point of view, this phenomenon can be regarded as a control system, which prevents expression of biodegradation capabilities when they are dispensable, thus improving the bacterial adaptation to its nutritional and energetic needs. This regulatory phenomenon is known as carbon catabolite repression (CCR) and can be exerted in different ways. Regulation of gene expression at both transcriptional and translational levels has been described in different biodegradation pathways [55]. CCR of *thn* genes has been demonstrated in TFA and TFB [14,38]. However, it is exerted by different carbon sources, which are metabolized via distinct central pathways, and through different mechanisms.

#### 4.2.1. Carbon Catabolite Repression in TFA

**Figure 4:**
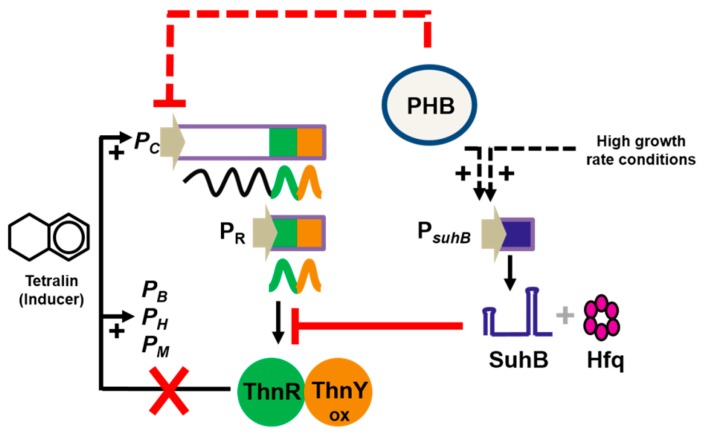
Elements involved in carbon catabolite repression in TFA. High growth rate conditions like growth on a preferential carbon source like βHB and the presence of PHB granules have a positive effect on *suhB* transcription. This sRNA, together with Hfq, affects negatively ThnR translation in those conditions impeding induction of *thn* promoters (*P_C_*, *P_B_*, *P_H_* and *P_M_*) in the presence of tetralin. The presence of PHB granules themselves have a negative effect on *thn* induction. Doted and solid lines represent unknown and known mechanism, respectively.

In TFA, the presence of βHB or fatty acids, such as sebacic acid, in the medium containing tetralin prevents the induction of *thn* genes [38]. Regarding the regulatory mechanisms, despite all the efforts invested in its hunt, like transposon mutagenesis or affinity binding assays, no transcriptional regulators responsible of CCR have been found in TFA. However, a clear connection between CCR and the energetic status of the cell has been demonstrated in this bacterium (Figure 4). Transposon mutants affecting in *phaC* gene, coding for a poly(3-hydroxybutyrate) synthase, are unable to synthetize the carbon storage PHB (polyhydroxybutyrate) granule and show a partial release of *thn* genes CCR [56]. Conversely to what happens in the wild-type, *phaC* mutants prematurely induce *thn* genes in the presence of βHB, although maximal induction is never reached. Homo- and heterologous complementation restores the wild type phenotype indicating that the amount of PHB granule is an internal signal of the energetic status of the cell that prevents *thn* genes expression in CCR conditions. However, CCR in TFA does not only rely on PHB synthesis.

Recently, a new element involved in CCR of TFA *thn* genes has been identified. A trans-encoded small non-coding RNA (sRNA), 61-nt long, named SuhB, and highly conserved in *Sphingopyxis* genus, was annotated in TFA genome and experimentally identified by dRNA-seq and Northern blot experiments [8,57]. SuhB deletion mutants exhibit *thn* gene de-repression in CCR conditions [57]. sRNAs are 50–250 bp long transcripts acting as bacterial riboregulators of mRNAs encoded elsewhere in the genome. The presence of hundreds of these molecules has been demonstrated in a range of bacteria post-transcriptionally controlling the expression of genes involved in a wide range of important processes, including cell division, responses to abiotic stimuli, quorum sensing and virulence [58,59]. sRNAs affect translation and/or stability of their mRNA targets and most of them are Hfq-dependent. Hfq is a RNA-binding protein that facilitates the binding of the sRNA to its target and/or protects it from degradation [60,61]. In TFA growing under high growth rate conditions, SuhB binds the 5’ UTR of *thnR* mRNA, thus negatively affecting its translation in an Hfq-dependent manner. As result, *thn* genes are silenced even if tetralin is present in the medium. The lack of SuhB or Hfq provokes a premature induction of *thn* genes in CCR conditions. However, only partial induction, even lower than that reached in *phaC* mutants, is achieved [56,57]. It has been demonstrated that *suhB* expression is regulated by the growth rate, being elevated at high growth rates, which would imply a high-energy status of the cells. In a *phaC* mutant, the absence of PHB granule could be detected as a low-energy status, *suhB* will not be transcribed and *thn* genes would be induced in the presence of tetralin. 

The high conservation of SuhB in other *Sphingopyxis* strains that do not bear *thn* genes suggests that this sRNA must be controlling other targets in these bacteria and that its involvement in the regulation of the tetralin degradation genes should have appeared more recently. This is another interesting example of how existing bacterial posttranscriptional regulatory mechanisms can be incorporated into the regulatory system of probably horizontally acquired metabolic pathways.

#### 4.2.2. Carbon Catabolite Repression in TFB

**Figure 5:**
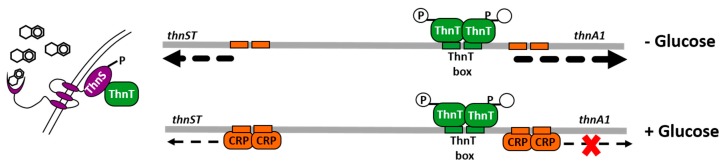
Elements involved in carbon catabolite repression in TFB. In the absence of glucose and presence of tetralin, phosphorylated ThnS transfer the P group to ThnT which binds the ThnT box and induces *thnA1* transcription. In the presence of glucose, a CRP-like protein binds CRP-like binding sites present at *thnS* and *thnA1* promoters provoking a reduction in *thnS* transcription and impeding *thnA1* transcriptional activation by phosphorylated ThnT.

CCR in TFB seems to be exerted in a more canonical way (Figure 5). In the presence of glucose, a FNR/CRP-like protein binds to the *thnS-thnA1* intergenic region [54]. A palindromic nucleotide sequence (CTGTGT-N6-TCACAG) similar to the *E. coli* CRP binding site was found overlapping the -10 and +1 sequences of *thnA1.* Thus, the binding of the FNR/CRP-like protein would prevent effective transcription of *thn* genes. In fact, point mutants in this sequence prevents binding of the FNR/CRP-like protein and provokes a partial relief of CCR of a *thnA1::gfp* translational fusion in TFB [54]. Partial de-repression could be due to the presence of a similar sequence at the *thnS* transcription start point to which the FNR/CRP could also bind, thus preventing *thnST* transcription. In fact, *thnS* expression, measured by RT-qPCR, is 5-fold higher in tetralin-grown cells than in glucose or glucose plus tetralin grown cells. The CCR regulatory protein of strain TFB would then act as a transcriptional repressor of *thn* genes in CCR conditions.

## 5. Concluding Remarks

The organic solvent tetralin is composed of an aromatic and an alicyclic ring, none of which is easily biodegraded in nature. The biodegradation pathways described to degrade alicyclic and aromatic rings are completely different and use different enzymatic activities. However, a similar strategy for tetralin biodegradation has evolved in unrelated bacteria by which a single enzymatic team, typically involved in biodegradation of one aromatic ring, is able to break open both rings of the tetralin in two subsequent steps, thus rendering products easily catabolised by many bacteria. Apparently, the *Rhodococcus* strain also uses part of this enzymatic team to degrade naphthalene, thus representing an interesting way of reutilising bacterial enzymatic activities for different purposes. Although the biodegradation strategy and the enzymatic activities are the same, elements regulating the expression of the *thn* genes are completely different in both strains, thus supporting the view that catabolic pathways and their regulatory systems evolve independently. Particularly worth highlighting is the exquisite and sophisticated *thn* gene regulation observed in strain TFA, in which three different regulatory circuits act together at transcriptional and posttranscriptional levels to optimise the gene expression levels to the environmental conditions. One of the circuits involves transcription activation of different promoters in a non-canonical way for a LysR-type regulator, which implies cooperative transcription from divergent promoters. The other circuit affecting transcription constitutes an unprecedented regulatory system aimed to prevent gratuitous induction by related molecules that are not substrate of the catabolic pathway. Finally, the third system uses a less abundant type of regulators, an sRNA, to control *thn* genes’ expression. Sophistication of these regulatory systems suggests that this biodegradation pathway has not been assembled recently as a response to human activity but bacteria might have been exposed to tetralin a long time ago, possibly as a component in the oil reservoirs.

The work presented here shows the usefulness of using a combination of different methodological approaches such as mutagenesis, mutant isolation, gene cloning, functional complementation, biochemical characterization and purification of enzymes, high throughput proteome analysis and chemical identification of metabolites to discover and characterize new mechanisms of microbial degradation. The knowledge gained substantially contributes to improving our understanding of important processes such as new metabolic capabilities and their regulatory mechanisms that control integrated physiological responses in bacteria other than the main models.

## Figures and Tables

**Table 1:** Homology between TFA and TFB proteins.

Protein	Funtion	TFA (aa)	TFB (aa)	Identity (%)/ Coverage (%)
ThnA1	tetralin dioxygenase, α-subunit	460	456	62/94
ThnA2	tetralin dioxygenase, β-subunit	181	182	59/95
ThnA3	ferredoxin	104	121	46/92
ThnA4	ferredoxin reductase	339	406	50/3
ThnB	cis-dihydrodiol dehydrogenase	270	270	57/100
ThnC	extradiol dioxygenase	304	299	65/97
ThnD	hydrolase	284	285	54/98
ThnE	hydratase	267	285	60/97
ThnF	aldolase	255	253	50/95
ThnG	aldehyde dehydrogenase	475	476	36/94
ThnH	CoA transferase	405		
ThnQ	pimeloyl-CoA synthase		706	
ThnJ	pimeloyl-CoA dehydrogenase, large subunit	397		
ThnK	pimeloyl-CoA dehydrogenase, small subunit	379		
ThnV	acyl-CoA dehydrogenase		383	
ThnL	enoyl-CoA hydratase and 3-hydroxyacyl-CoA dehydrogenase	695	295*	42/28
ThnW	3-hydroxyacyl-CoA dehydrogenase		286*	32/38
ThnI	acetyl-CoA acetyl transferase	402	389	55/99
ThnM	TonB-dependent protein	723		
ThnN	glutaryl-CoA dehydrogenase	394		
ThnO	flavoprotein, α-subunit	249		
ThnP	flavoprotein, β-subunit	312		
ThnU	putative sterol transfer protein		131	

TFA proteins were used as query in BlastP analysis with default parameters. *Identity of ThnL^TFB^ and ThnW^TFB^ to ThnL^TFA^ N and C-terminal part, respectively.
